# A True Random Number Generator Design Based on the Triboelectric Nanogenerator with Multiple Entropy Sources

**DOI:** 10.3390/mi15091072

**Published:** 2024-08-25

**Authors:** Shuaicheng Guo, Yuejun Zhang, Ziyu Zhou, Lixun Wang, Zhuo Ruan, Yu Pan

**Affiliations:** Faculty of Electrical Engineering and Computer Science, Ningbo University, Ningbo 315211, China; 2311100345@nbu.edu.cn (S.G.); zzyeric@foxmail.com (Z.Z.); wanglixun@tom.com (L.W.); 2311100240@nbu.edu.cn (Z.R.); 2311100235@nbu.edu.cn (Y.P.)

**Keywords:** triboelectric nanogenerator, true random number generators, multi-entropy sources, differential algorithm

## Abstract

The triboelectric nanogenerator (TENG) has the potential to serve as a high-entropy energy harvester, enabling the self-powered operation of Internet of Things (IoT) devices. True random number generator (TRNG) is a common feature of encryption used in IoT data communication, ensuring the security of transmitted information. The benefits of multiplexing TENG and TRNG in resource-constrained IoT devices are substantial. However, current designs are limited by the usage scenarios and throughput of the TRNG. Specifically, we propose a structurally and environmentally friendly design based on the contact–separation structure, integrating heat fluctuation and charge decay as entropy sources. Furthermore, filtering and differential algorithms are recommended for data processing based on TENG characteristics to enhance randomness. Finally, a TENG-based TRNG is fabricated, and its performance is verified. Test results demonstrate a random number throughput of 25 Mbps with a randomness test pass rate approaching 99%, demonstrating suitability for resource-constrained IoT applications.

## 1. Introduction

The increasing prevalence of electronic devices and the expansion of instrumentation in the industrial sector have led to a demand for more effective interaction with these devices. Implementing the Internet of Things (IoT) could effectively address these needs. Two critical issues that demand urgent attention are ensuring the long-term reliable operation of IoT devices [1] and secure connections [2] among mutually independent IoT devices. IoT devices should always be situated in natural environments remote from the power grid, such as forests [3,4], oceans [5,6], and mountains [7,8], due to the necessity for detection. Efforts are underway to extend the life of IoT devices by minimizing energy consumption or harvesting energy from the environment. Furthermore, a lack of encryption can lead to the leakage of critical information on the device [9,10]. Information security depends on key protection, where key strength depends on the entropy source used for generation. The prevailing approach is to use the physical unpredictability of phenomena to generate true random numbers, thereby ensuring robust key generation and information security.

**Figure 1:**
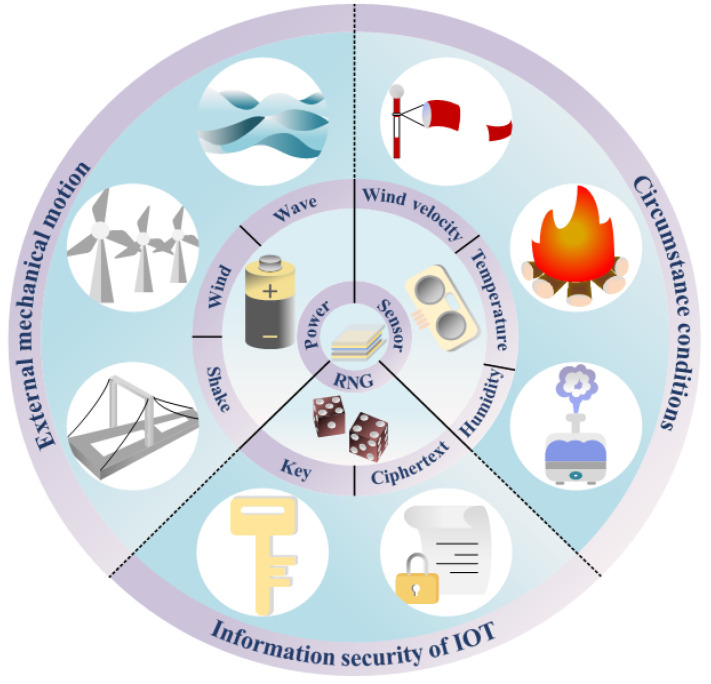
The TENG is an energy harvester, sensor, and random number generator.

The triboelectric nanogenerator (TENG), a novel energy harvesting device introduced by Z.L. Wang and colleagues in 2012 [11], capitalizes on triboelectricity and electrostatic induction to harvest high-entropy energy. Its unique energy harvesting capabilities and output characteristics make it ideal for synergizing with IoT systems. As shown in Figure 1, TENGs can be used as power sources [12,13,14,15] to power the data processing and communication module. TENG-based sensors are used to obtain environmental information [16,17,18,19], while TENG-based True random number generators (TRNGs) are used to generate random numbers. Thus, with TENGs, a fully self-powered IoT system can be realized to solve resource-constrained problems for IoT applications. The current TENG-based TRNG uses chaotic motion induced by wind [20,21,22] or raindrops [23] as the entropy source; the above-mentioned TRNG requires a specialized design of the structure based on the characteristics of the entropy source, so it cannot be used directly in the existing TENG structure. In addition, wind and rain, as entropy sources, can only be used at specific times and locations, which does not guarantee the operational efficiency of the TRNG, making throughput and security compromised.

To address these limitations, we propose an enhanced TRNG system based on the TENG. Firstly, our approach introduces a random number generation scheme utilizing the traditional contact–separation mode of TENGs. This allows the TRNG design to be used in the existing contact–separation mode of TENGs. Secondly, the scheme is based on the charge decay on the surface of triboelectric materials and the heat fluctuation on electrodes during TENG motion. This approach fully extends the adaptation of the TRNG in space and time due to the wide availability of entropy sources, and the quality of the random numbers is improved due to the joining of multiple entropy sources for random number generation. Finally, signal processing uses high-pass filtering and differential algorithms to eliminate periodic components and further enhance randomness. The resulting system is not only unrestricted in the use of occasions and structural design, but also achieves a random number generation frequency of 25 Mbps, and the resulting randomness performance is comparable to previous studies.

## 2. Methods

This section examines the principles of TENG and TRNG, with particular emphasis on the sources of randomness, namely, the entropy source. In addition, it also describes the function of filtering and differencing algorithms and randomness indicators. Additionally, it introduces the process of TENG electricity generation, crucial to designing methods that ensure high randomness, and the sieving process between random and non-random signals.

### 2.1. Principles of TENG

Energy generation in the TENG is based on the Maxwell displacement current. The contact and separation motion of the TENG causes a change in the polarization field of the triboelectric charge on the surface, thus generating a polarization current. This is the fundamental source of power that enables the TENG to output energy [24]. The mathematical expression can be written as

(1) $$J_{S}=\frac{\partial P_{S}}{\partial t},$$

In general, the external output performance of TENGs is of greater interest to researchers. The TENG capacitance model is a fundamental theory for carrying out circuit design, output performance enhancement, and other purposes. The potential difference between the two Cu electrodes of a TENG, the output voltage, can be given by

(2) $$V=-\frac{1}{C(x)}Q+V_{OC}(x),$$

where *Q* is the charge on the Cu electrode, which changes with the contact and separation motion of the TENG. *C*(*x*) is the equivalent capacitance of the TENG, and *V*_OC_(*x*) is determined by the charge density and the capacitance of triboelectric materials [25]. The TENG proposed in this paper is of the contact–separation type. Its contact area is considerably larger than the maximal separation distance of the TENG; thus, it can be approximated as an infinite parallel plate capacitor.

(3) $$C(x)=\frac{\varepsilon_{0}S}{d+x(t)},$$

(4) $$V_{OC}(x)=\frac{\sigma x(t)}{\varepsilon_{0}},$$

(5) $$d=\sum_{i=1}\frac{d_{i}}{\varepsilon_{i}}.$$

**Figure 2:**
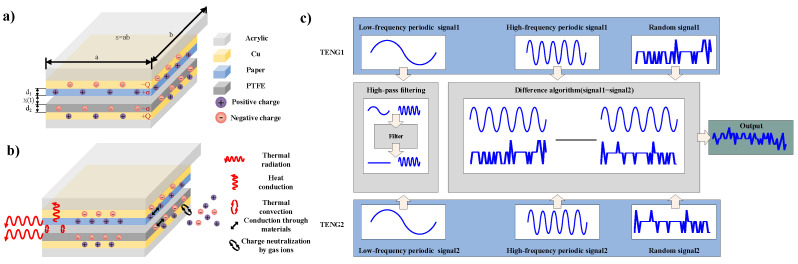
(**a**) Materials and structural parameters of TENG; (**b**) entropy sources of TENG; (**c**) principles of differential algorithm and high-pass filtering.

In the above equations, *ε*_0_ represents the permittivity of vacuum; *S* represents the surface area of the electrode; *d* is the equivalent thickness of the triboelectric material, in which *d_i_* and *ε_i_* represent the thickness and relative capacitance from the top to the bottom of each layer of material, in addition to the substrate; *x*(*t*) represents the distance between the positive and negative triboelectric materials; and *σ* represents the charge density of the triboelectric material. The relationship between the parameters and the TENG is illustrated in Figure 2a. It can be demonstrated that the distance between the triboelectric materials is altered due to the movement of the TENG, resulting in an alternating voltage on an external circuit.

### 2.2. Analysis of Sources of Randomness

The random data on the TENG voltage signal have been analyzed as having two main entropy sources, the charge decay on the triboelectric materials and the heat fluctuation on the electrodes during motion. The charge density on the surface of the triboelectric materials increases as a result of TENG contact and saturates very quickly. Conversely, the surface charge density slightly decreases during separation due to processes such as conduction through materials, surface conduction, and charge neutralization by ions in the gas [26].

(6) $$\frac{d\sigma_{v}(\vec{r},t)}{\mathrm{dt}}=j_{0}e^{\frac{-t}{\rho_{v}\varepsilon }},$$

(7) $$\frac{d\sigma_{S}(\vec{r},t)}{\mathrm{dt}}=0,$$

(8) $$\frac{d\sigma_{G}(\vec{r},t)}{\mathrm{dt}}=\frac{e}{\mathrm{dA}(\vec{r})}\int \frac{\mathrm{dn}}{\mathrm{dt}}dV_{.}$$

The change in charge density for conduction through materials, surface conduction, and charge neutralization by ions in the gas are represented by Equations (6)–(8), respectively, where *r* is the position vector, *t* is time, j_0_ is the initial current density, *ρ*_v_ is the volume resistivity of the materials, and *ε* is the dielectric constant. The change in charge density for surface conduction is 0 because the electric field can be approximated as perpendicular to the surface, pointing from the upper half of the TENG to the lower half, and the tangential component of the surface is 0. e is the amount of charge per unit of charge, *A*(*r*) represents the surface area of the TENG, *dn*/*dt* represents the rate at which the charge on the surface of the TENG is neutralized by the ions in the gas, and *V* represents the volume of the air affected. Through electrostatic induction, the charge density on the electrodes is affected by the change in charge density on the triboelectric materials mentioned above, which is manifested as a small voltage fluctuation on the external circuit.

Furthermore, the contact movement of the upper and lower parts of the TENG results in a rise in the temperature of the surface of the triboelectric materials. The higher temperature of the triboelectric materials causes heat to be transferred to the electrodes and the acrylic substrate through the process of heat conduction. In addition, the triboelectric materials are accompanied by heat convection and heat radiation. The change in heat on the electrodes is also expressed as a small voltage fluctuation on the external circuit [27,28], which can be described by the following equations:

(9) $$\bar{V^{2}}=\int \mathrm{RGd}\upsilon ,$$

(10) G=4kT.

*R* is the resistive part of the impedance, *υ* is the frequency, k is the Boltzmann constant, and *T* is the absolute temperature. The impact of entropy sources on randomness can be illustrated in Figure 2b.

Regarding the two entropy sources mentioned above, we can consider that the charge decay follows the normal distribution E_1_∼N (μ_1_, σ_1_^2^) and that the heat fluctuation also follows the normal distribution E_2_∼N (μ_2_, σ_2_^2^). Under the condition of independence, according to the superposition of the normal distribution, the two entropy sources still follow the normal distribution E_1_ + E_2_∼N (μ_1_ + μ_2_, σ_1_^2^ + σ_2_^2^ ) after superposition. The superposed normal distribution has a larger variance, which means that the combined effect of the two entropy sources leads to a larger range of variability and uncertainty, and the corresponding randomness is also improved.

### 2.3. Filtering and Differential Algorithms

The randomness of TRNGs is attributed to the changes in charge density on the triboelectric materials and the fluctuations in heat during movement. Due to the relatively low amplitude of the random voltage generated by the entropy sources, it is typically masked by the voltage signal generated by the TENG movement, so no random signal can be detected.

Filtering technology selects frequency components in the signal by weakening the amplitude of unrelated components. As a result, the output data contain only the required frequency components. The voltage generated by the TENG motion is a low-frequency periodic signal, while the voltage generated by the entropy sources is evenly distributed across all frequencies. Random signal, the voltage generated by the entropy sources, can be extracted by suppressing the low-frequency components and retaining the high-frequency components through the technique of high-pass filtering.

The differencing algorithm subtracts one input signal from another, thereby eliminating common components. High-frequency periodic signals mainly include electromagnetic interferences (EMIs) from the external environment, which inevitably impact the randomness by being coupled with the test system. However, When EMIs are coupled to the Cu electrodes of two TENGs close to each other, the resulting waveforms and frequencies are identical. The voltage generated by TENG1 is subtracted from the voltage generated by TENG2 through the differential algorithm. The EMIs existing in both TENGs can be eliminated, but the random signals of the two TENGs are completely different and still exist after differential operation. The principles regarding the filtering and differencing algorithms are depicted in Figure 2c.

### 2.4. Indicators of Randomness

Autocorrelation is a crucial indicator of randomness. Its primary function is to ascertain the degree of similarity between a sequence and itself at a given time delay. The higher the similarity, the stronger the periodic component of the signal and therefore the worse the randomness. Autocorrelation can be quantified by the autocorrelation coefficient *ρ*_xy_, which varies between −1 and 1. The closer the value to 1 or −1, the worse the randomness of the signal. Conversely, the closer to 0, the better the randomness of the data, meaning that even if we have access to all the data from a previous period, we cannot accurately predict the current or future data.

The FIPS 140-2 test is a metric for evaluating the quality of random numbers and contains four tests, namely, the Monobit Test, the Poker Test, the Runs Test, and the Long Runs Test. Each test performs a pre-determined calculation on input random sequences and provides results. The test sequence will be considered to have passed the test and be random only if the result falls within the corresponding reference range. However, these test items and the length of the test sequence are limited. To ensure the randomness of the test sequence, tests such as Frequency within a Block and Serial are also performed. The SP800-22 Random Number Test Suite from the National Institute of Standards and Technology (NIST) provides a more comprehensive test of the randomness of the data, comprising a total of 15 tests. The number of data for each test item must be selected under the specific characteristics of each test item. To inform this selection, we consulted the relevant literature [29,30], which suggests the following selection, as shown in Table 1. The length is defined as the number of data points entered at a time, while the bitstreams represent the times of tests.

### 2.5. TENG Production and Electricity Generation Process

**Figure 3:**
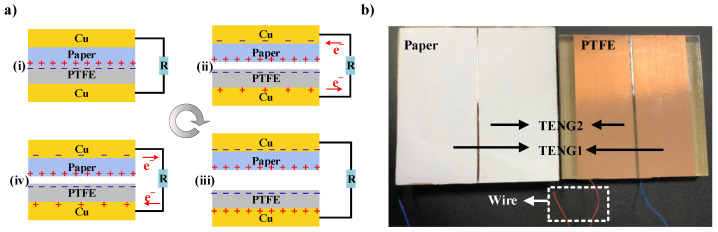
(**a**) The electricity generation process of TENG in contact–separation mode; (**b**) The TRNG consisting of TENG1 and TENG2.

The TRNG presented in this paper is based on two TENGs in traditional contact–separation mode. The structure of the TENG is depicted schematically in Figure 3a, and Figure 3b illustrates the physical model of the two TENGs that constitute the TRNG. About the selection of materials for the TENG, paper was chosen as the positive triboelectric material, polytetrafluoroethylene (PTFE) was chosen as the negative triboelectric material, copper foil was chosen as the electrode, and acrylic was chosen as the substrate. The paper substrate, with dimensions of 10 cm by 4 cm, had a thickness of 100 nm. The PTFE layer, also measuring 10 cm by 4 cm, was 80 nm thick. The Cu electrodes, matching the size of the PTFE at 10 cm by 4 cm, exhibited a thickness of 60 nm. The acrylic, larger in area at 10 cm by 8.5 cm, had a significantly greater thickness of 3 mm. Given the prevalence of contemporary radio communication technology, EMI is inevitable [31]. To mitigate the impact of EMI on the performance of the TRNG, it is proposed that two TENGs be placed close to each other. This configuration allows the voltage generated by the EMI on the electrodes of the two TENGs to be nearly identical, which can be eliminated by differential operation as mentioned in Section 2.3.

The electricity generation process of the TENG is illustrated in Figure 3a. In stage 1, the triboelectric materials of the TENGs are in contact with each other. The triboelectric series [32] indicates that PTFE is readily capable of gaining electrons and is therefore negatively charged, whereas paper relatively easily loses electrons and is thus positively charged. A favorable matching of gained and lost charges ensures that the respective surfaces of triboelectric materials carry more equal and opposite charges when they are in contact. In stage 2, the TENGs begin to separate, resulting in the induction of charges on the electrodes. The upper part of the TENG induces negative charges on the Cu electrode, while the lower part induces positive charges. The corresponding electron flow on the external circuit is from the bottom to the top.

In stage 3, as the separation distance reaches a maximum, the corresponding maximum charges are induced on the electrodes. In stage 4, the TENGs approach each other from the maximum separation distance. The negative charge of the upper electrode starts to decrease, while the positive charge of the lower electrode decreases. The direction of the electron flow on the external circuit is from the top to the bottom. The charge continues to move until the two TENGs come back into contact again. The TENGs periodically perform the aforementioned reciprocating movement, and charge transfers between the upper and lower electrodes and subsequently in the external circuit to produce alternating current output.

### 2.6. The Process of Generating Random Numbers

**Figure 4:**
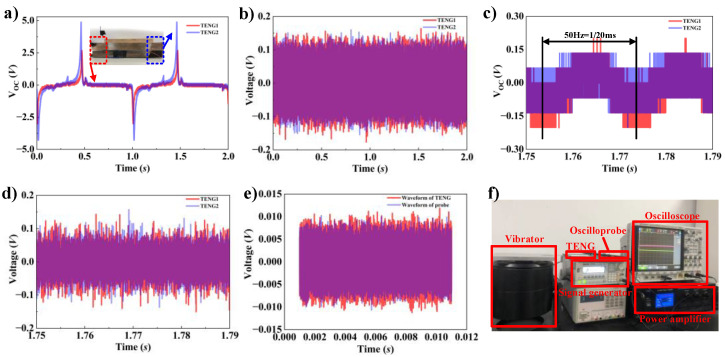
(**a**) The waveforms of TENG1 and TENG2; (**b**) the waveforms after high-pass filtering; (**c**) enlarged diagrams of the waveforms of TENG1 and TENG2; (**d**) enlarged TENG waveforms after high-pass filtering; (**e**) voltage from heat fluctuations; (**f**) experimental platform and test equipment.

The red voltage waveform belongs to TENG1, while the blue voltage waveform belongs to TENG2, as illustrated in Figure 4a. It can be concluded from the figure that the two voltage waveforms are not identical. While the frequency and shapes of the two waveforms are identical, there is a significant difference in their amplitudes and phases. This is because the TENGs are inherently tilted due to inevitable design flaws, which results in the one closer to the tilted direction producing a higher voltage and having a phase difference. Nevertheless, this phenomenon does not affect the randomness, due to the fact that the differences can be filtered out by using high-pass filtering as illustrated in Section 2.3. Our filter is designed by MATLAB software and is a 99-order Butterworth high-pass filter with a cutoff frequency of 70 KHz. Because the voltage frequency of the TENGs is lower than 70 KHz, the voltage will be suppressed by the filter, and components with corresponding frequencies higher than 70 KHz will be retained. The filtered result is shown in Figure 4b.

The amplification of the voltage waveform from the TENGs, as illustrated in Figure 4c, reveals periodic characteristics with a frequency of approximately 50 Hz. This phenomenon is attributed to power line interference, due to the proximity of the oscilloscope and TRNG to the power transmission line. The voltage waveforms after high-pass filtering can be observed in Figure 4d, where the periodic voltages caused by power line interference have been eliminated. The following components should exist in each voltage after filtering: the voltage caused by the heat fluctuation and the voltage arising from charge decay on the triboelectric materials. Additionally, the high-frequency EMIs from the surrounding environment, including mobile phone communication signals, alternating current transmission harmonic interference, wireless broadcasting signals, and so on, couple into the oscilloscope. The presence of EMI has the potential to affect the randomness of the output. However, it can be mitigated by the differential algorithm as illustrated in Section 2.3, thereby enhancing the randomness of the remaining signal.

When the oscilloscope probe does not measure any object, the oscilloscope still detects a voltage, which is high-pass-filtered to obtain a blue waveform, as shown in Figure 4e, which is the noise voltage generated by the oscilloscope and the probe. When the oscilloscope and probes are connected to the moving TENGs for detection of the voltage generated by the TENGs, the voltage is high-pass-filtered to obtain a red waveform. It is not difficult to find that the amplitude of the red waveform is larger than that of the blue waveform most of the time, which indicates that the TENGs generate additional voltage. We speculate that this part of the voltage is generated by the heat fluctuation on the TENGs, because the movement of the TENGs will make the surfaces of the materials collide, and part of the kinetic energy in this process will be converted into the heat energy on the surfaces of the materials, which will generate a voltage according to Equation (9).

## 3. Results

This section focuses on detailing the experimental platform used, the performance of the random number generation scheme, the quality of the generated random numbers, factors influencing randomness, and applications of these random numbers.

### 3.1. Experimental Platforms

As illustrated in Figure 4f, the oscilloscope utilized in this experiment was a Keysight DSOX4024A, ranging in bandwidth from 200 MHz to 1.5 GHz. The probe utilized was the Keysight N2894A, with a magnification of 10×, an input impedance of 10 MΩ, and a maximum sampling frequency of 700 MHz. The shaker was YMC VT-500. The signal generator employed was a RIGOL DG1022U, and the power amplifier used was a YMC LA-800. The upper end of the TENGs served as the active component fixed to the shaker, while the lower one represented the stationary component fixed to a stationary platform. Driven by the shaker, the moving part periodically contacted and separated from the stationary part, thereby generating alternating current voltage output as mentioned in Section 2.5.

### 3.2. Factors Affecting Randomness

**Figure 5:**
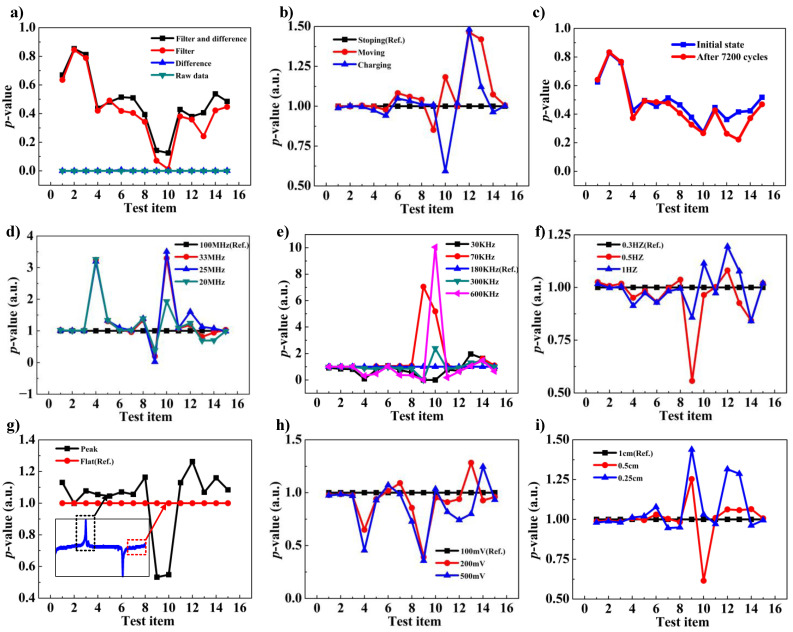
(**a**) Influence of difference and filtering algorithms on randomness; (**b**) influence of triboelectric material wear on randomness; (**c**) entropy sources analysis of TRNG and their influence on randomness; (**d**) influence of oscilloscope sampling frequency on randomness; (**e**) influence of filtering frequency on randomness; (**f**) influence of TENG motion frequency on randomness; (**g**) influence of waveform sampling position on randomness; (**h**) influence of oscilloscope amplitude resolution on randomness; (**i**) influence of TENG motion amplitude on randomness.

To enhance the performance of the TENG-based TRNG with multi-entropy sources, this section will analyze in detail the impact of factors on the randomness of the TRNG. The experimental results are presented in Figure 5a–i. The range of numbers from 1 to 15 along the *x*-axis represents the 15 test items from NIST-SP800-22, with the correspondence between these numbers and the items detailed in Section 2.4 Table 1. The *y*-axis displays the *p*-values associated with each item.

The experimental results obtained from Figure 5a illustrate that the randomness of the raw data and the data processed only by the difference algorithm is very poor. However, if the data are filtered by the high-pass filter, the randomness can be considerably improved. The differencing algorithm is applied to the data after high-pass filtering, as shown by the black broken line; at least nine items of the *p*-values are significantly improved. These results demonstrate that the filtering and difference algorithms do eliminate the low-frequency and high-frequency periodic components and improve randomness, as described in Section 2.3.

Given the unfeasibility of extracting entropy source signals individually, a method of controlling variables is demonstrated to indirectly verify the randomness of two entropy sources. Initially, the TENGs are kept stationary for acquiring the first set of data. Then, the TENGs are driven to test the second set of data. Finally, TENGs are stopped to test the third set of data immediately.

The first set of data uses heat fluctuation as the entropy source, while the second and third sets of data use heat fluctuation and charge decay as the entropy sources. To make the comparison more obvious, we divide the *p*-values of each set by the reference *p*-values of the first set of data, and the results are shown in Figure 5b, where the black, red, and blue broken lines correspond to the first, second, and third sets of data, respectively. The *p*-values of the blue broken line are larger than those of the black broken line, where larger *p*-values correspond to better randomness. The blue broken line possesses better randomness because the surfaces of the triboelectric materials of the TENGs still retain a portion of charges after stopping the motion [33], and the decay of charges will act as another entropy source to enhance randomness.

The *p*-values corresponding to the red broken line are the largest of all cases, which means the best randomness. Compared with the TENG state corresponding to the blue broken line, the TENGs corresponding to the red broken line are constantly undergoing contact–separation motion, and a part of the kinetic energy during the motion is converted into thermal energy, which improves the intensity of heat fluctuation and produces stronger randomness. Through the above analysis, it can be concluded that both heat fluctuation and charge decay as entropy sources can generate randomness, and the joint effect of the two entropy sources improves the randomness.

Regarding the effect of material wear on randomness, the experimental results are displayed in Figure 5c, in which the blue broken line represents the randomness corresponding to the TENGs in the initial state, and the red broken line represents the randomness corresponding to the TENGs after running 7200 cycles continuously. It can be seen that compared with the initial state, there is a decrease in *p*-values after 7200 cycles, but the decreased *p*-values are still much higher than 0.01, which proves that the random numbers generated by the TRNG still have relatively good randomness and that the TENG-based TRNG has an excellent ability to run for a long time.

Then, to investigate the impact of the sampling frequency on the randomness of the TRNG, the highest sampling frequency of the oscilloscope probe is set to 700 MHz, and the sampled frequency of the signal cannot exceed 140 MHz, which is one-fifth of the highest sampling frequency. Consequently, the sampling frequencies for comparison in this experiment are 20 MHz, 25 MHz, 33 MHz, and 100 MHz, respectively. As illustrated in Figure 5d, the *p*-values for all sampling frequencies are divided by the reference *p*-values, which belong to 100 MHz. It can be demonstrated that from 100 MHz, the randomness of the TRNG increases as the sampling frequency decreases, reaching an optimum at 25 MHz, after which a decrease in randomness occurs as the filtering frequency is further reduced to 20 MHz.

Next, we investigate the impact of the high-pass filter cutoff frequency on randomness, as illustrated in Figure 5e. The experimental results indicate that the highest randomness level is achieved at a cutoff frequency of 70 KHz, with a decline observed at the cutoff frequency of 30 KHz. Moreover, increases in the cutoff frequency to 180 kHz, 300 kHz, and 600 kHz result in corresponding reductions in randomness. It may be attributed to a hypothesis that the upper frequency limit of the non-random signal is around 70 KHz, and when the cutoff frequency is exactly 70 KHz, the non-random signal is just completely filtered out, whereas when the cutoff frequency is smaller, the unfiltered periodic component has an effect on the final randomness, and when the cutoff frequency is larger, the random signal is filtered out, so that the final randomness decreases. The impact of the operating frequency of the TENGs on the randomness is illustrated in Figure 5f. As the operating frequency increases from 0.3 Hz to 0.5 Hz and 1 Hz, the *p*-values remain relatively constant and do not exhibit a linear change in regularity, which aligns with the theory that the operating frequency does not influence the output voltage of the TENG.

Furthermore, it is essential to investigate the impact of the sampling position of the waveforms on randomness. During operation, the TENGs undergo the cycle of motion and station. Based on the analysis of the entropy sources, it can be concluded that the performance of the random numbers generated is superior when the TENGs are moving. The voltage waveforms of TENGs are shown in the lower-left corner of Figure 5g, and the motion corresponds to the peaks, while the station corresponds to the flat part. The *p*-values corresponding to the stationary state are taken as a reference, and it is observed that the *p*-values of the moving state are almost always greater, indicating that the randomness is better when the TENGs are moving. The results are consistent with the theoretical predictions.

Additionally, the effect of the vertical resolution of the oscilloscope on randomness is explored, as illustrated in Figure 5h. As resolution increases, it is more difficult to capture the small voltage caused by heat fluctuations and charge decay, so the randomness decreases consistently as vertical resolution increases from 100 mV to 200 mV and 300 mV. Finally, the effect of TENGs motion amplitude on the generated randomness was explored, as shown in Figure 5i. As the TENG motion amplitude decreases from 1 cm to 0.5 cm and 0.25 cm, the randomness continuously increases. This phenomenon can be attributed to the fact that the voltages of the TENGs are reduced in magnitude as the amplitude of motion decreases. This allows for the easier detection of random signals. Based on this property of TENGs, we believe that TENGs are suitable for use in a self-sourced TRNG system. When the external excitation is strong and the movement of the TENGs is large, the TENGs are used for generating electrical energy and storing the energy in an energy storage device such as a capacitor. When the external excitation is weak and the movement of the TENGs is small, the TENGs are used as a TRNG, and the electrical energy stored in the capacitor is used to power data acquisition and processing modules.

### 3.3. Results of Randomness

**Figure 6:**
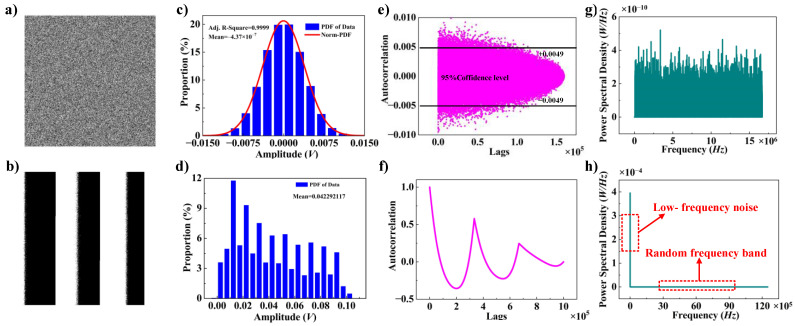
(**a**) Binary image of random signal; (**b**) binary image of periodic signal; (**c**) statistical distribution situation of random signal; (**d**) statistical distribution situation of periodic signal; (**e**) autocorrelation of random signal; (**f**) autocorrelation of a periodic signal; (**g**) power spectral density of random signal; (**h**) power spectral density of periodic signal.

By examining the impact of the aforementioned factors on the randomness of the TRNG, it can be demonstrated that when the sampling frequency of the oscilloscope is 25 MHz, the cutoff frequency of the high-pass filter is 70 KHz, the TENGs movement frequency is 0.5 Hz, the peak portion of the voltage waveform is captured, the vertical resolution of the oscilloscope is 100 mV/div, and the maximum movement amplitudes of the TENGs are 0.25 cm, the most optimal quality of random numbers is achieved. To guarantee the dependability of the randomness of the acquired data, the random numbers are subjected to a series of randomness verifications, including binary image, statistical distribution, autocorrelation, and power spectral density (PSD), the results of which are presented in Figure 6.

The binary image of the TENG waveform signal is depicted in Figure 6a. The binary image, measuring 1000 × 1000, represents a visual method for assessing randomness. The distribution characteristics of the graph demonstrate that resulting random numbers are completely stochastic, as there is no discernible regular shape arrangement. For comparison, the original TENG waveform data, which has not been processed by filtering and differencing algorithms, are converted into binary imagen as shown in Figure 6b. A comparison of Figure 6a with Figure 6b indicates that the randomness of the data processed by the filtering and differencing algorithms is significantly better and that the two algorithms play a vital role in enhancing the randomness.

The blue bar graph in Figure 6c represents the statistical distribution law of the amplitude of the waveform generated by the TENGs. The red curve is obtained by coupling the blue bar graph with a normal distribution curve. The closer the Adj. R-Square is to 1, the greater the degree of coupling. From this figure, it can be observed that the data in general follow the normal distribution law. The majority of the data are concentrated around 0 V. The statistical test of the data indicates that their mean value is −4.37 × 10^−7^, which is very close to 0. Additionally, the Adj. R-Square is equal to 0.9999, which is very close to 1, suggesting that the statistical distribution of these data is very close to the normal distribution. Values greater than 0 are converted to 1, while values lower than or equal to 0 are converted to 0. In this manner, the voltages are converted to binary data, ensuring that the resulting 0s and 1s remain unbiased in terms of the total number. The original data in Figure 6d do not exhibit symmetry and cannot be coupled to a normal distribution, so the resulting 0s and 1s appear regular.

The autocorrelation of the data after processing is illustrated in Figure 6e. The *y*-axis of the figure indicates the autocorrelation coefficient between the data before and after a delay. A larger coefficient indicates that the data between and after the delay are more similar. The *x*-axis is the amount of delay of the data. The test is conducted by using 160,000 data points. When the data are delayed by 160,000 data points, the data and the delayed data become completely staggered in time, and the autocorrelation coefficients become zero. This figure indicates that there is no discernible autocorrelation because the autocorrelation coefficients |*ρ*_xy_| are all below 0.01, which is in accordance with the rule of thumb for interpreting the size of a correlation coefficient [34]. This rule suggests that the autocorrelation between the data before and after the delay is relatively weak, with |*ρ*_xy_| lying within the range of 0 to 0.2, which is almost negligible. Conversely, as illustrated in Figure 6f, the data exhibit a strong autocorrelation, with |*ρ*_xy_| generally above 0.5 in the first 16 W of data.

The PSDs of the waveform data after processing can be observed in Figure 6g, where the PSDs are all approximately 3 × 10^−10^ W/Hz in different frequency components. This is analogous to the PSD plot of white noise, which is a highly desirable random signal. It can be concluded that the waveform data generated by this TENG should also exhibit ideal random characteristics to white noise. As illustrated in Figure 6h, The PSD of the original data shows that the low-frequency components have high energy, while the high-frequency components have comparatively lower energy. This difference can be quantified as a 10^6^-fold ratio between the two components. This indicates that the high-frequency components are largely subsumed by the low-frequency components. The low-frequency components generated by the periodic motion of the TENG exhibit a lack of randomness, which is a fundamental reason for the poor randomness of the original data.

The outcomes obtained in the NIST-SP800-22 test are presented in Table 2. The information regarding the settings of length and bitstreams in the test is consistent with the descriptions in Table 1. The first column indicates the names of the test items, the third column represents the percentages of random numbers that pass the test, and the second column represents the calculated average *p*-values. The results shown in the last column demonstrate that the random numbers generated by the TRNG successfully pass all test items and exhibit high levels of randomness. This is evidenced by the fact that the pass rate of tests is 95% or above, with an average value of approximately 99%.

Furthermore, as shown in Table 3, the results from the FIPS-140-2 test are presented. The names of test items are located in the first and fourth columns of the table. The first number before the name of each item represents the object of the item record. When the object is 0, only 0s in the sequence are recorded. Subsequently, equations and inequalities are presented after the names of the items, representing the numbers of consecutive occurrences of the aforementioned 0s, where the counting process will continue until there is an interruption in consecutive 0s. These are only counted if the specified conditions are met, and the count results are provided in the second and fifth columns. The random numbers generated by the TRNG pass the randomness tests in FIPS-140-2, which serves as further evidence that the random numbers obtained are sufficiently random.

Compared with the state-of-the-art TENG-based TRNGs, the design in this paper has advantages in terms of throughput, structural adaptability, and applied environment, as shown in Table 4. This work utilizes heat fluctuation and charge decay as the entropy sources and shows almost the same randomness test pass rate as past studies. While ensuring random number quality, this TRNG design shows better reliability and adaptability.

### 3.4. Encryption and Decryption

**Figure 7:**
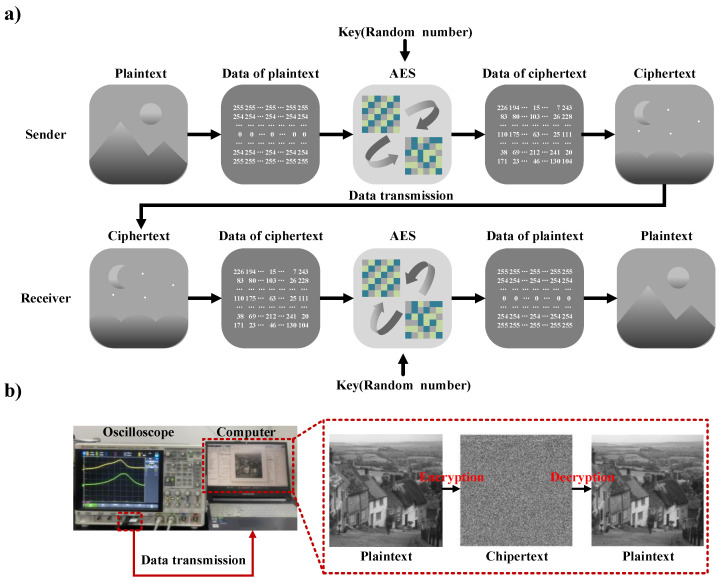
(**a**) Principle of encrypted communication via AES; (**b**) encryption and decryption experiments using random numbers generated by TRNG as keys.

The random numbers generated by a TRNG can be applied to the IoT for the encryption of information during communication. As an illustrative example, consider the advanced encryption standard (AES). In this case, the information of the sender must be transmitted to the receiver while promising that others cannot obtain the meanings of the information. Therefore, the information is encrypted by the AES before it is sent. In this encryption process, random numbers are used as a key to encrypt the information. The information before encryption and that after encryption are unrelated to each other. If a third party receives the encrypted information but lacks the key, they cannot ascertain the meaning of the information. Only the recipient who possesses the key in advance can decrypt and acquire the meaning through the key. The principle of encrypted communication via AES is illustrated in Figure 7a.

The encryption and decryption experiments are illustrated in Figure 7b. The random numbers generated by the TRNG are acquired by the oscilloscope and then transferred to the computer by a USB flash disk. On the computer, the random numbers serve as the key in the AES encryption and decryption process. As evidenced by the results, the image loses its original meaning after encryption and the meaning can be fully recovered after decryption. In the Video S1, experimental processes of encryption and decryption are shown, including steps like data acquisition and random verification. This proves the ability of the random numbers generated by our TRNG in IoT information security.

## 4. Discussion

In conclusion, this paper presents a multi-entropy-source fusion, high-throughput TRNG based on TENGs as a solution to existing problems like poor structural adaptability and low throughput. The TRNG primarily fuses heat fluctuations and charge decay as the entropy sources, thereby achieving a structurally and environmentally friendly TRNG design. Furthermore, filtering and differencing algorithms are employed to optimize randomness after fully considering the characteristics of the TENGs and the entropy sources. The potential influences on randomness were experimentally explored, and the results demonstrate that the sampling frequency, filtering frequency, and sampling location have a significant impact on the randomness of the TRNG. The experimental results demonstrate that a random number throughput of 25 Mbps can be achieved while passing the NIST-SP800-22 and FIPS-140-2 tests. This renders the TENG-based TRNG suitable for use in the field of information encryption for IoT devices. However, there are still some shortcomings in this article; for example, the roles of differencing algorithms for various types and intensities of EMIs have not been explored in depth, and research on reducing the power consumption of the TRNG has not been carried out in detail. Therefore, we will push forward the research of these elements in the next step to realize an ultra-low-power or even self-sourced TRNG.

## Figures and Tables

**Table 1:** Length and bitstreams selection for each NIST-SP800-22 test item.

Test item	Length/ Bitstreams	Test Item	Length/ Bitstreams
(1) Frequency	10,000/300	(8) Non-Overlapping Templates	1,000,000/3
(2) Block Frequency	10,000/300	(9) Overlapping Templates	1,000,000/3
(3) Cumulative Sums	10,000/300	(10) Universal	1,000,000/3
(4) Runs	10,000/300	(11) Approximate Entropy	10,000/300
(5) Long Run of Ones	10,000/300	(12) Random Excursions	1,000,000/3
(6) Rank	38,912/77	(13) Random Excursions Variant	1,000,000/3
(7) Spectral DFT	65,536/45	(14) Linear Complexity	1,000,000/3
		(15) Serial	10,000/300

**Table 2:** NIST-SP800-22 tests for TRNG.

Testing Results of NIST-SP800-22 (α = 0.01; 3,000,000 bits)			
Statistical Test	Proportion	*p*-Value	Results
Frequency	100%	0.6673	Pass
Block Frequency (m = 2000)	100%	0.84548	Pass
Cumulative Sums	100%	0.79368	Pass
Runs	98%	0.43757	Pass
Long Runs of Ones	98%	0.51336	Pass
Rank	98%	0.52694	Pass
Spectral DFT	97%	0.4829	Pass
Non-overlapping Templates (m = 10)	97%	0.46344	Pass
Overlapping Templates (m = 10)	100%	0.32542	Pass
Universal	100%	0.15535	Pass
Approximate Entropy (m = 8)	98%	0.46179	Pass
Random Excursions	95%	0.46902	Pass
Random Excursions Variant	100%	0.55797	Pass
Linear Complexity (M = 500)	100%	0.42915	Pass
Serial (m =10)	99%	0.49929	Pass

**Table 3:** FIPS 140-2 tests for TRNG.

Testing Results of FIPS 140-2 (20,000 Bits)					
Statistical Test	Number	Results	Statistical Test	Number	Results
1 Monobit	9985	Pass	0 Runs ≥ 6	187.0	Pass
0 Monobit	10,015	Pass	0 Long Runs	0.0	Pass
Poker	8.521	Pass	1 Runs = 1	2407.0	Pass
0 Runs = 1	2475.0	Pass	1 Runs = 2	1293.0	Pass
0 Runs = 2	1217.0	Pass	1 Runs = 3	628.0	Pass
0 Runs = 3	599.0	Pass	1 Runs = 4	303.0	Pass
0 Runs = 4	318.0	Pass	1 Runs = 5	155.0	Pass
0 Runs = 5	146.0	Pass	1 Runs ≥ 6	156.0	Pass
			1 Long Runs	0.0	Pass

**Table 4:** Performance comparison with state-of-the-art TRNGs.

	[20]	[21]	[22]	[23]	This Work
Entropy source(s)	Wind	Wind	Wind	Raindrop	Multi-entropy sources
Throughput	20 Kbps	10 Kbps	20 Kbps	1.6 Kbps	25 Mbps
Structural adaptability	Weak	Weak	Weak	Weak	Strong
Applied environment	Wind speed of 5 m/s	Wind speed of 12.8 m/s	Wind speed of 4 m/s	Rainy day	Almost all
Randomness	NIST: 99.4%	NIST: 99.6%	NIST: 99.5%	Autocorrelation: 99%	NIST: 98.6%

## Data Availability

The raw data supporting the conclusions of this article will be made available by the authors on request.

## Supplementary Materials

The following supporting information can be downloaded at: https://www.mdpi.com/article/10.3390/mi15091072/s1, Video S1: Supplementary movie.
